# Neuritin 1 promotes retinal ganglion cell survival and axonal regeneration following optic nerve crush

**DOI:** 10.1038/cddis.2015.22

**Published:** 2015-02-26

**Authors:** T P Sharma, Y Liu, R J Wordinger, I-H Pang, A F Clark

**Affiliations:** 1 North Texas Eye Research Institute, University of North Texas Health Science Center, Ft. Worth, TX 76107, USA; 2 Department of Cell Biology & Immunology, University of North Texas Health Science Center, Fort Worth, TX 76107, USA; 3 Department of Pharmaceutical Sciences, College of Pharmacy, University of North Texas Health Science Center, Ft. Worth, TX 76107, USA

## Abstract

Neuritin 1 (Nrn1) is an extracellular glycophosphatidylinositol-linked protein that stimulates axonal plasticity, dendritic arborization and synapse maturation in the central nervous system (CNS). The purpose of this study was to evaluate the neuroprotective and axogenic properties of Nrn1 on axotomized retinal ganglion cells (RGCs) *in vitro* and on the *in vivo* optic nerve crush (ONC) mouse model. Axotomized cultured RGCs treated with recombinant hNRN1 significantly increased survival of RGCs by 21% (*n*=6–7, *P*<0.01) and neurite outgrowth in RGCs by 141% compared to controls (*n*=15, *P*<0.05). RGC transduction with AAV2-CAG–hNRN1 prior to ONC promoted RGC survival (450%, *n*=3–7, *P*<0.05) and significantly preserved RGC function by 70% until 28 days post crush (dpc) (*n*=6, *P*<0.05) compared with the control AAV2-CAG–green fluorescent protein transduction group. Significantly elevated levels of RGC marker, RNA binding protein with multiple splicing (Rbpms; 73%, *n*=5–8, *P*<0.001) and growth cone marker, growth-associated protein 43 (Gap43; 36%, *n*=3, *P*<0.01) were observed 28 dpc in the retinas of the treatment group compared with the control group. Significant increase in Gap43 (100%, *n*=5–6, *P*<0.05) expression was observed within the optic nerves of the AAV2–hNRN1 group compared to controls. In conclusion, Nrn1 exhibited neuroprotective, regenerative effects and preserved RGC function on axotomized RGCs *in vitro* and after axonal injury *in vivo*. Nrn1 is a potential therapeutic target for CNS neurodegenerative diseases.

Central nervous system (CNS) trauma and neurodegenerative disorders trigger a cascade of intrinsic and extrinsic cellular events resulting in regenerative failure and subsequent damage to neurons.^1, 2, 3, 4, 5^ The intrinsic factors include deregulation in growth-promoting factors, apoptotic factors, intracellular signaling molecules and trophic factors.^6^ Similarly, the extrinsic factors correlate to growth inhibition due to inhibitory cues^3, 7, 8, 9, 10, 11, 12, 13^ that include myelin and myelin associated inhibitors, glial scarring,^5, 14^ slow clearance of axonal debris,^7^ incorrect development of neuronal projections^6^ and CNS inflammation.^15, 16^ Progressive degeneration of mature retinal ganglion cells (RGCs) has been associated with loss of trophic support,^8, 9^ detrimental inflammatory processes/immune regulation^10, 11^ and apoptotic effectors.^9, 12, 13, 15, 17^

After injury, mammalian RGC axons show only a short-lived sprouting response but no long-distance regeneration through the optic nerve (ON).^16^ Glial responses around the affected area are initiated by injured CNS axons.^18^ Axons undergoing Wallerian degeneration are surrounded by astrocytes that upregulate glial fibrillary acidic protein (*Gfap*) expression and these reactive astrocytes contribute to trauma-induced neurodegeneration.^19^ Glial scarring inhibits axonal transport after ON crush (ONC)^5, 14^ decreasing transport of proteins involved in neuroprotection and synaptic plasticity. Regenerative failure is a critical endpoint of these destructive triggers culminating in neuronal apoptosis^3, 20, 21^ and inhibition of functional recovery. Intrinsic factors affecting axonal regeneration after CNS injury are crucial for recovery and thus, dysregulation of genes involved in axonal plasticity and outgrowth can prove detrimental to the neuronal recovery.^22, 23, 24^

Current neuroprotection approaches include promoting survival of RGCs by intraocular injections of recombinant factors like ciliary neurotrophic factor (CNTF) and peripheral nerve (PN) transplantations *in vitro*^25^ and *in vivo* after injury.^26^ Studies performed with glial cell-line-derived neurotrophic factor and neurturin protect RGCs from axotomy-induced apoptosis.^27^ Further, in the ON injury model, RGC survival was promoted after deletion of CCAAT/enhancer binding protein homologous protein^28^ and enhanced regeneration observed with co-deletion of kruppel-like factor 4 (*Klf4*) and suppressor of cytokine signaling 3 (*Socs3*).^29^ Intraocular administration of neurotrophin-4 (NT-4) and brain-derived neurotrophic factor (BDNF) after ON transection has also exerted neuroprotective effects on axotomized RGCs. In addition, PNs transplanted adjacent to ONs, *ex vivo* PN grafts with lenti-viral transduced Schwann cells, and stimulation of inflammatory processes have strong pro-regenerative effects on injured RGCs.^26, 30, 31, 32, 33^

In addition, using adeno-associated-*virus* (AAV) therapy, AAV mediated expression of CNTF in bcl2 overexpressing transgenic mice increases cell viability and axonal regeneration,^34^ whereas BDNF promotes survival of RGCs.^35^ Likewise, experiments with AAV–BDNF, –CNTF and –growth-associated protein 43 (GAP43) have shown that AAV–CNTF was the most crucial for promoting both long-term survival and regeneration.^36^ The positive effects of CNTF are observed mainly through simultaneous deletion of both PTEN and SOCS3^37^ and the concurrent activation of mTOR and STAT3 pathways.^38^ Although CNTF shows robust increase and sustained axon regeneration in injured ONs of rodents, it causes axonal misguidance and aberrant growth.^39^ Furthermore, it has been shown that CNTF acts as a chemoattractant. CNTF administration onto autologous PN grafts transplanted within transected ON increased regeneration, but these effects were significantly reduced after removal of macrophages from this site.^40^ In addition, the effects of CNTF using PN grafts at ON transection sites are further subject to debate, as previously it has been shown that Ad-CNTF injections preserved RGC axons but did not induce regeneration of axotomized RGCs.^41^ Thus, other studies have addressed RGC survivability and axonal regeneration with CNTF and other growth factors,^35, 36^ but most trophic factors affect neuronal survival and regeneration differentially.

Previous studies targeting neuronal apoptosis by overexpressing intrinsic growth factors, inhibiting apoptosis and enhancing regeneration in CNS trauma models have established that a multifactorial approach is required for successful and long-lasting therapeutic outcomes.^6, 36^ Current gaps still exist for a key gene that could effectively target neuroprotection, enhance neuron regeneration and sustain neuronal function.

One key gene implicated in neuronal plasticity is Neuritin 1 (*Nrn1*), also known as candidate plasticity gene 15. It has multiple functions and was first identified and characterized when screening for candidate plasticity genes in the rat hippocampal dentate gyrus activated by kainate.^42, 43, 44^ *Nrn1* is highly conserved across species^45^ and translates to an extracellular, glycophosphatidylinositol-linked protein (GPI-linked protein), which can be secreted as a soluble form. Nrn1 stimulates axonal plasticity, dendritic arborization and synapse maturation in the CNS.^46^ During early embryonic development, Nrn1 promotes the survival of neural progenitors and differentiated neurons,^47^ while later in development it promotes axonal and dendritic growth and stabilization, allowing maturation and formation of synapses.^43, 46, 48^ In the adult brain, Nrn1 has been correlated with activity-dependent functional plasticity^45, 49^ and is expressed in post mitotic neurons.

*Nrn1* may be a crucial gene for neuroprotection and regeneration because growth factors such as nerve growth factor (NGF), BDNF and NT-3 as well as neuronal activity can potentiate the expression of *Nrn1*.^44, 50^ In addition, we reported that *Nrn1* mRNA expression appears to be biphasic after ON axonal trauma, indicating a transient attempt by RGCs at neuroprotection/neuroregeneration in response to ONC injury.^51^ The dynamic regulation of *Nrn1* coupled with neurotrophic effects may promote axonal regeneration in the CNS. To overcome CNS trauma, a new therapy geared towards neuroprotection and effective axonal regeneration is required to enhance a future multifactorial approach. The purpose of this study is to evaluate the therapeutic effects of Nrn1 in mouse RGC cultures as well as in the mouse ONC model. We have identified a distinct neuroprotective and regenerative strategy that prevents neurodegeneration after ON injury. AAV2–hNRN1 expression vectors partially rescued RGCs from apoptosis, maintained RGC function, and initiated regeneration of injured axons.

## Results

### Recombinant hNRN1 increases RGC neuroprotection and neurite outgrowth *in vitro*

Our mixed retinal cell culture was characterized by using markers (Supplementary Table S1) of different retinal cell types (Supplementary Figures S1a–h). We used two different markers to identify RGCs within these cultures. We observed a higher percentage of RNA binding protein with multiple splicing (Rbpms) positive cells (36±1%, *n*=8) compared with brain-specific homeobox/POU domain protein 3A (Brn3a; 17±1%, *n*=8; Supplementary Figure S1i). In addition, the majority of cells were RGCs, although we observed other retinal cell types (amacrine cells, astrocytes, Müller cells, microglia, bipolar cells; Supplementary Figures S1a–h) within the mixed culture.

**Figure 1:**
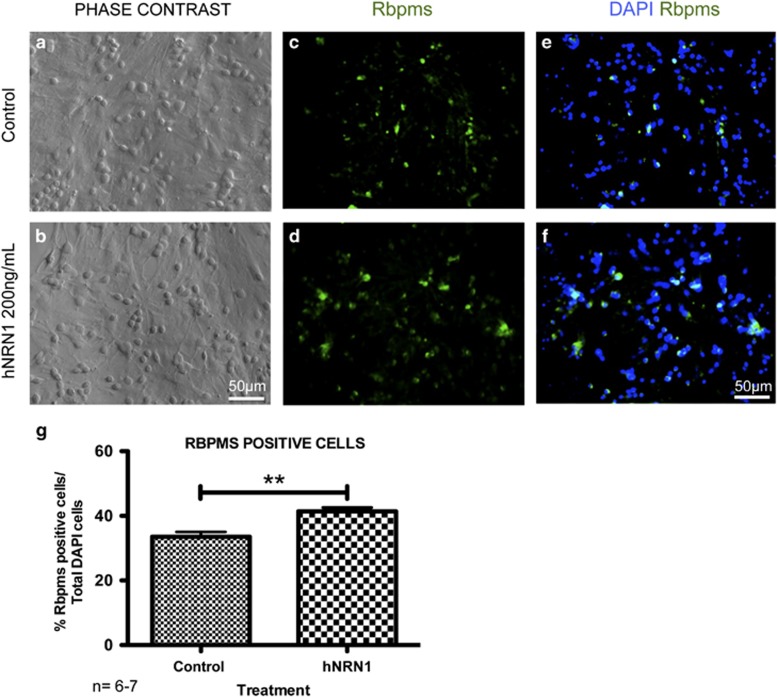
Increased survival of axotomized RGCs by recombinant human NRN1 *in vitro*. Dissociated retinal cultures were treated with vehicle (control) or recombinant hNRN1 (200 ng/ml; treatment) for 10 days in culture. Phase contrast images of (**a**) control and (**b**) treatment. Fluorescence micrographs of dissociated RGCs immunolabeled with Rbpms (**c**) control and (**d**) treatment and merged with DAPI (**e**) control and (**f**) treatment. Blue staining indicates DAPI labeled nuclei with green immunostaining for Rbpms expression. Scale bar=50 *μ*m. Photomicrographs were captured at 400 × original magnification. (**g**) Quantification of Rbpms stained RGCs per well. Values represent the mean of five images per well from three independent experiments per group. Data are presented as mean±S.E.M. Statistical significance between treatment conditions determined by unpaired Student's *t*-test, **P<0.01, *n*=6–7

To test the neuroprotective and regenerative effects of Nrn1 *in vitro,* we exposed the cells to medium without growth factors and treated the axotomized RGCs in the mixed culture with recombinant hNRN1 (200 ng/ml). After treatment, phase contrast images of cultures showed robust increase in cells (Figures 1a and b). NRN1 treatment significantly increased the number of Rbpms-positive RGCs by 21% (41±1, mean±S.E.M., *n*=7, RGCs in NRN1-treated cultures *versus* 34±1 RGCs in control cultures, *n*=6; *P*<0.01) (Figures 1c–g).

**Figure 2:**
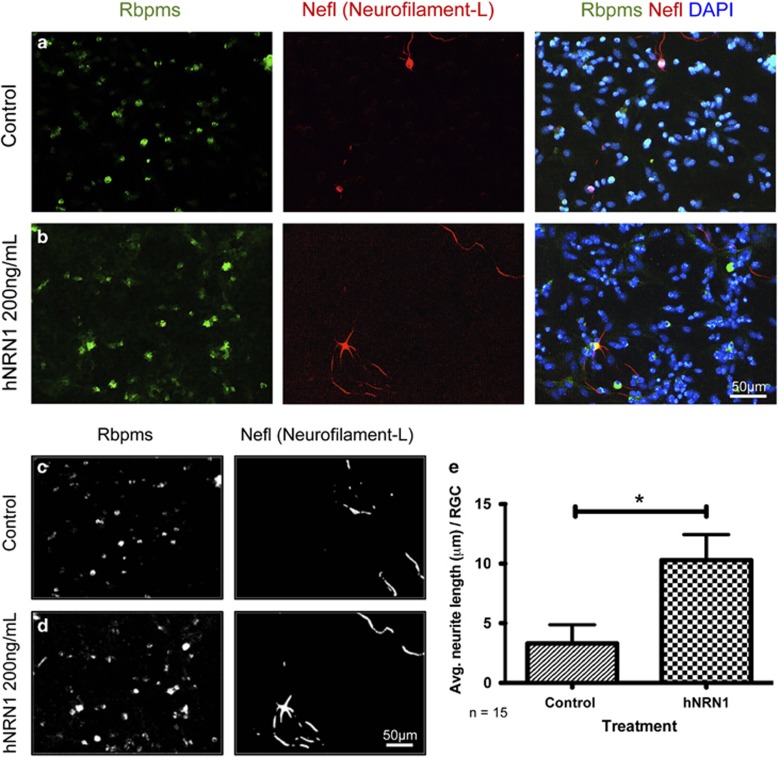
Recombinant human NRN1 increases neurite outgrowth in axotomized RGCs *in vitro*. Dissociated retinal cultures were treated with vehicle (control) or recombinant hNRN1 (200 ng/ml) for 10 days in culture. Fluorescence micrographs of neurite outgrowth in dissociated RGCs immunolabeled with Rbpms, Nefl and DAPI in (**a**) control and (**b**) NRN1-treated cells. Blue staining indicates DAPI labeled nuclei, green immunostaining for Rbpms and red for Nefl. Processed Rbpms-positive nuclear and Nefl neuronal tracings in (**c**) control and (**d**) NRN1-treated cells. Scale bar=50 *μ*m. Photomicrographs were captured at 400 × original magnification. Quantification of average length of (**e**) neurites per RGC as described in **a**–**d**. Values represent the mean of total Rbpms-positive cells and total neurite length (*μ*m) for four random images captured in 39 100 *μ*m^2^ area for 15 wells from 4 independent experiments per group. Data are presented as mean±S.E.M. Statistical significance between treatment conditions determined by unpaired Student's *t*-test, *P<0.05, *n*=15

Compared with the control, recombinant NRN1 increased RGC neurite outgrowth (Figures 2a and b). Neurite and nuclear tracings (Figures 2c and d) also showed a higher neurite tracing pattern in RGCs after treatment. Quantification of total neurite length per 39, 100 *μ*m^2^ area showed significantly increased neurite outgrowth in RGCs by 141% (*P*<0.05) with NRN1 treatment (16.22±6.25 *μ*m, *n*=15) compared to non-treated control cells (6.73±2.71 *μ*m, *n*=15) (Figure 2e).

### AAV2-mediated overexpression of *hNRN1*

**Figure 3:**
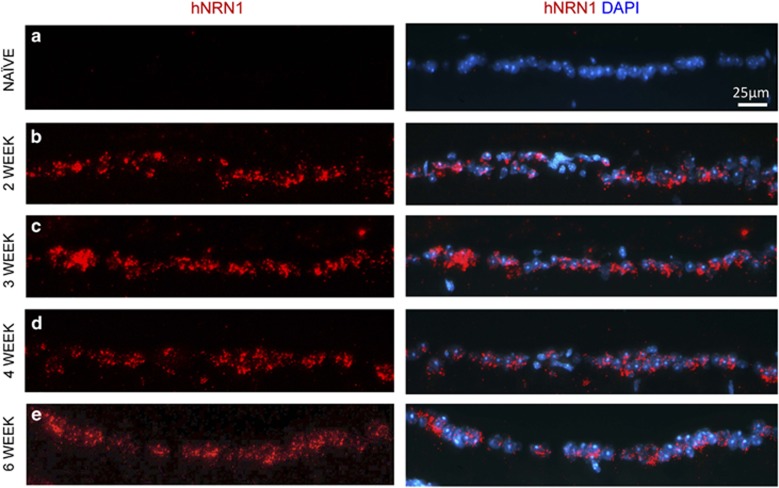
Increased retinal *NRN1* mRNA expression after AAV2-mediated *NRN1* overexpression. BALB/cJ mice received intravitreal injections of AAV2–hNRN1. *In situ* hybridization was performed on retinal sections probed for *NRN1. NRN1* signals are present in the GCL. Retinal sections from (**a**) naïve, (**b**) 2 weeks, (**c**) 3 weeks, (**d**) 4 weeks and (**e**) 6 weeks after intravitreal injection. Blue staining indicates DAPI labeled nuclei, all panels represent red for *hNRN1* in the RGC layer. Scale bar=50 *μ*m, *n*=4. Photomicrographs were captured at 400 × original magnification

We performed *in situ* hybridization to test mRNA expression of human *NRN1* in AAV2–hNRN1-injected mouse eyes. No expression was observed in the naïve retinas (Figure 3a). Two weeks after injection, we observed robust selective staining predominantly in the ganglion cell layer (GCL) suggesting that RGCs and/or displaced amacrine cells are the major retinal cell types expressing *NRN1* (Figure 3b). The expression was maintained through 6 weeks after injection (Figures 3c–e). AAV2 is selective but not specific to RGCs, and although increased expression of *NRN1* can be observed within the GCL, AAV2 can also transduce other cell types such as bipolar and photoreceptor cells.^35, 52^ The *NRN1* mRNA expression is evident within other layers of the retina (Supplementary Figures S2a and b).

**Figure 4:**
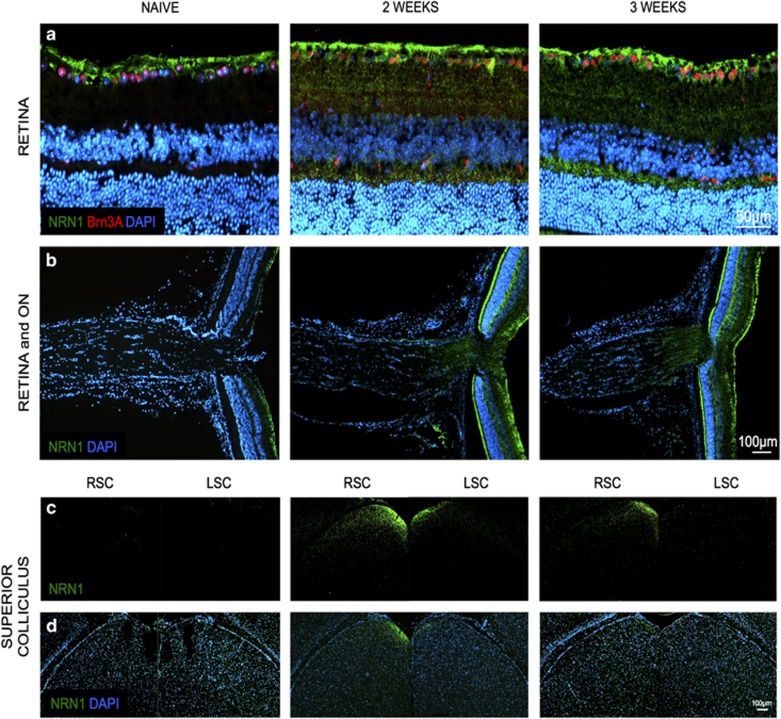
Increased expression of hNRN1 *in vivo* after intravitreal injection of AAV2-CAG–hNRN1. BALB/cJ mice received intravitreal injections of AAV2–hNRN1 and were harvested at naïve, 2 and 3 weeks after injection. Fluorescence micrographs show overexpression of hNRN1 in longitudinal (**a**) retinal, (**b**) retina and ON and (**c** and **d**) SC coronal sections. Green=NRN1; red=Brn3a; blue=DAPI. Time point post injection indicated on top of the panel. RSC (right contralateral superior colliculus) and LSC (left ipsilateral superior colliculus). Scale bar=50 *μ*m (**a**), 100 *μ*m (**b**–**d**), *n*=4. Photomicrographs were captured at 400 × and 100 × original magnification

Overexpression of NRN1 was also confirmed by immunohistochemical (IHC) staining. Increased hNRN1 was specifically detected as early as 2 weeks in the nerve fiber layer and the cells of the GCL (Figure 4a). Since NRN1 is a secreted protein,^43^ slight expression was also observed within the inner and outer plexiform layers (Figure 4a). Similarly, within the ON there was increased expression 2 weeks after intravitreal injection (IVT) compared with the naïve non-injected control eyes, and immunoreactivity was maintained at 3 weeks (Figure 4b). Comparable to NRN1 expression, our control vector (AAV2–green fluorescent protein (AAV2–GFP)) also presented robust expression at 3 weeks post IVT in the retina and ON (Supplementary Figures S3a and b).

To determine whether the transgene proteins were anterogradely transported through the visual pathway, we tested expression of GFP and hNRN1 in the superior colliculus (SC). Visual signals are transmitted from the retina to the lateral geniculate nucleus and SC through the RGC axons. In rodents, most retinal inputs project to the contralateral SC with minor innervation to the ipsilateral SC.^53^ We observed increased NRN1 staining in the contralateral SC and faint staining within the ipsilateral SC, which was still maintained at 3 weeks (Figures 4c and d). The AAV2–GFP virus also showed marked GFP expression at 3 weeks in the contralateral SC compared with the naïve animals (Supplementary Figures S3c and d).

### AAV2–hNRN1 mediated neuroprotection and axonal regeneration after axonal trauma

**Figure 5:**
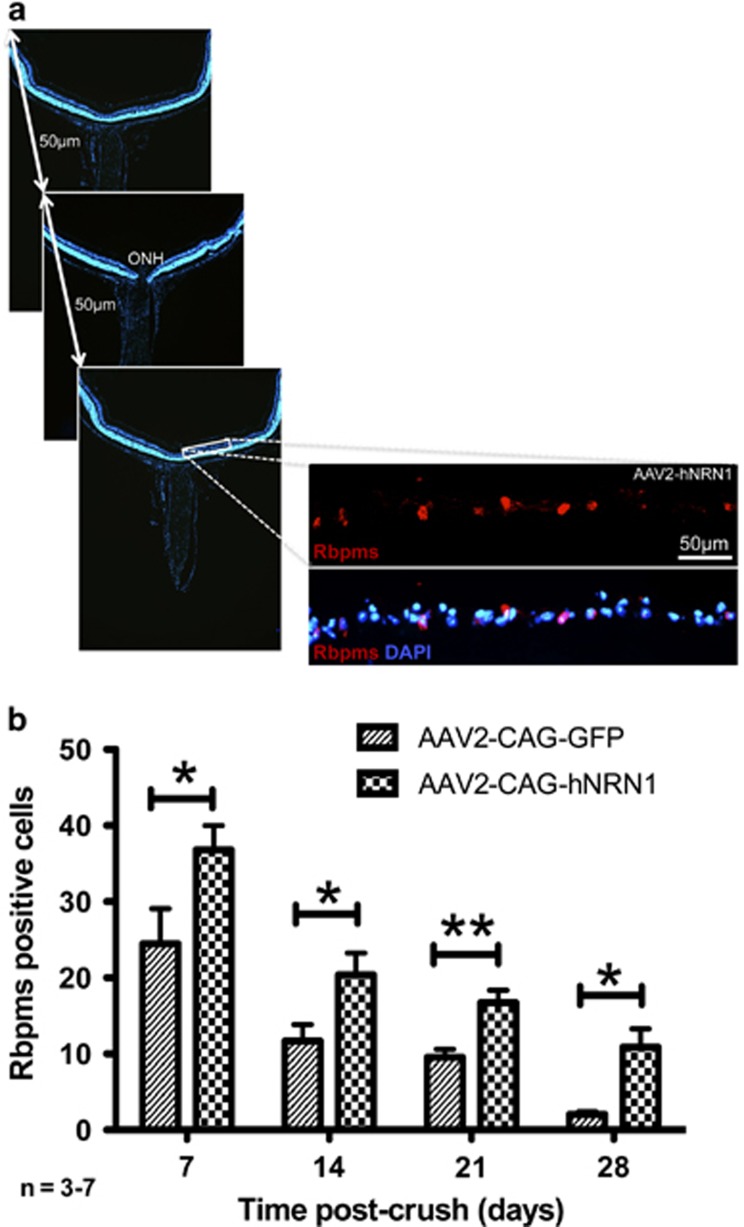
AAV2-mediated delivery of *NRN1 in vivo* promoted and sustained survival of axotomized RGCs. Animals were intravitreally injected with either AAV2–GFP or AAV2–hNRN1. Two weeks later, animals were subjected to ONC and whole eyes harvested at 7, 14, 21 and 28 days post crush. (**a**) Representation of retinal tissue sections counted from ora serrata to ora serrata in the central region of each mouse retina (six sections/eye). Fluorescent micrographs of Rbpms and Rbpms with DAPI images at 14 days post crush show representative images captured at each point throughout the whole retina to count the Rbpms-positive cells (400 × magnification, Scale bar=50 *μ*m). (**b**) Quantification of Rbpms-positive cells. Naïve retinas presented a mean of 168±23 cells (*n*=4). Data are presented as mean±S.E.M. Statistical significance between treatment conditions determined by unpaired Student's *t*-test, *P<0.05, **P<0.01, *n*=3–7

To analyze RGC survival, retinal sections were examined for Rbpms immunoreactivity from ora serrata to ora serrata through the central region of the mouse eye (Figure 5a). Quantitative analysis of Rbpms revealed more surviving RGCs in the AAV2–hNRN1-injected eyes compared with the control AAV2–GFP eyes. There was a significant increase in RGCs in the AAV2–hNRN1 group compared with the AAV2–GFP group at all time points post crush. At 7 days post crush (dpc), a significant increase was observed (54%, *P*<0.05) in the AAV2–hNRN1 group (37±3, *n*=7) compared with the AAV2–GFP controls (24±5, *n*=5) and a significant 66% increase (*P*<0.05) at 14 dpc within the AAV2–hNRN1 eyes (20±3, *n*=7) *versus* the controls (12±2, *n*=6). At 21 dpc, there was a significant increased survival of 70% (*P*<0.01) in the AAV2–hNRN1 group (17±2, *n*=7) compared with the control AAV2–GFP group (10±1, *n*=6) which was further enhanced by fivefold at 28 dpc showing a significant (*P*<0.05) increase at 28 dpc in the AAV2–hNRN1 (11±2, *n*=7) *versus* the control AAV2–GFP eyes (2±0, *n*=3) (Figure 5b).

Gap43 is a classical marker of axonal regeneration.^54, 55, 56^ Cytoskeletal proteins transport Gap proteins to the injured end of the axon, where they are incorporated into the membranes of growth cones.^57, 58^ An active RGC regenerative state was induced by overexpression of hNRN1 in our ONC model as seen by increased immunoreactivity of Gap43 within the retina and ONs after crush. Gap43 immunoreactivity in our control group showed expression patterns similar to those observed previously in other axonal injury studies.^55, 59^

**Figure 6:**
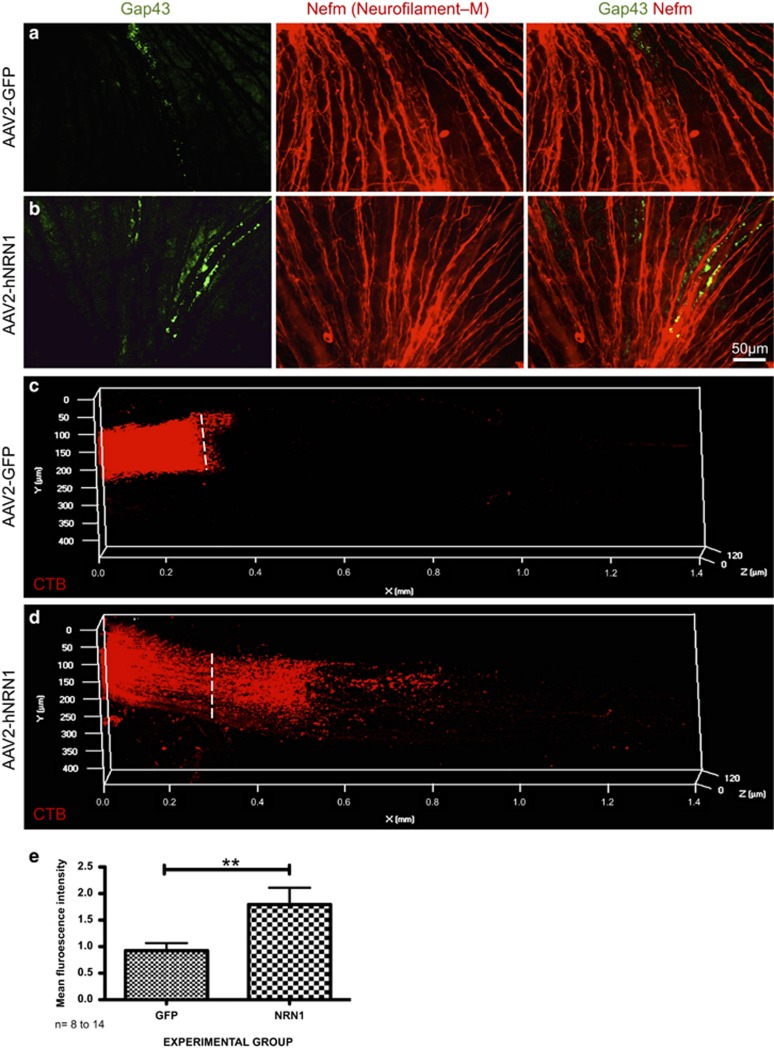
Retinal ganglion cell axonal growth is stimulated by AAV2-CAG–hNRN1 in the ONC model. Animals were intravitreally injected with either AAV2–GFP or AAV2–hNRN1. Two weeks later, animals were subjected to optic nerve crush and whole eyes harvested at 28 days post crush. Fluorescence micrographs of retinal flat mount sections immunolabeled with Gap43 and Nefm (**a**) AAV2–GFP and (**b**) AAV2–hNRN1. In retinal flat mounts, red indicates Nefm and green is Gap43. Three-dimensional construction of whole ONs injected with CTB-594: (**c**) AAV2–GFP and (**d**) AAV2–hNRN1. Red staining in whole ONs indicates CTB-594 labeled axons. Dotted line represents crush site within each ON image. Scale bar=50 *μ*m (**a** and **b**, *n*=5), (**c** and **d**, *n*=4–8). Photomicrographs were captured at 400 × (**a** and **b**) and 200 × (**c** and **d**) original magnification. (**e**) Quantification of mean fluorescence intensity of CTB-594. Data are presented as mean±S.E.M. Statistical significance between treatment conditions determined by unpaired Student's *t*-test, **P<0.01, *n*=8–14

In retinal flat mounts of AAV2–hNRN1-injected ONC mice, we observed elevated expression of Gap43 within the RGC axons compared with the AAV2–GFP control group at 28 dpc (Figures 6a and b). The increased expression of Gap43 signifies increased regeneration due to overexpressed NRN1 compared to the controls.

To further analyze the extent of regeneration past the crush site in both AAV2–GFP and AAV2–hNRN1-injected groups, cholera toxin B subunit (CTB) was intravitreally injected in both groups to measure the anterograde transport of the protein past the crush site. Increased transport of CTB was detected posterior to the lesion site in the AAV2–hNRN1 group at 28 dpc, which was not apparent within the AAV2–GFP control group as observed in the 3D presentations of ONs from both groups (Figures 6c and d). Quantification of the mean fluorescence intensity showed a significant increase in intensity past the crush site within the NRN1 group over control (*P*<0.01) (Figure 6e). This indicates a marked regenerative effect due to hNRN1 overexpression allowing effective CTB transport through the RGC axons past the crush site.

**Figure 7:**
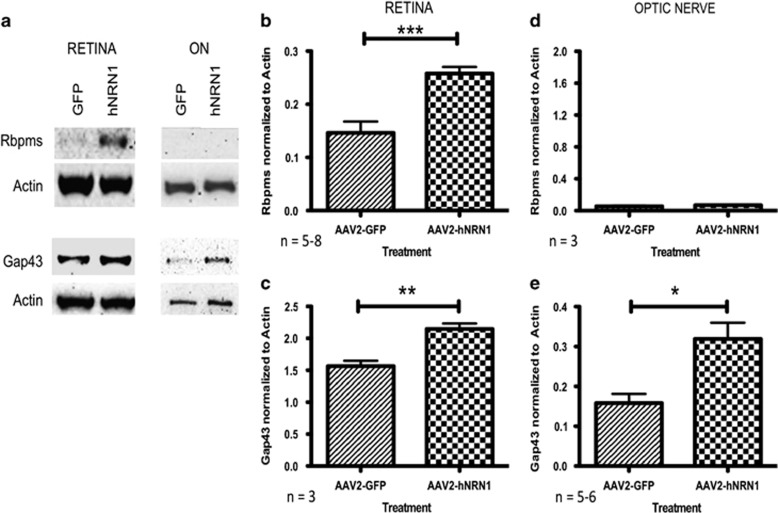
AAV2-CAG–hNRN1 mediated survival and regeneration of RGCs in the *in vivo* ONC model. Animals were intravitreally injected with either AAV2–GFP or AAV2–hNRN1. Two weeks later, animals were subjected to ONC and retinas and ONs harvested 28 days post crush. (**a**) Western blot analysis of retinal and ON lysates from AAV2–GFP and AAV2–hNRN1 animals using antibodies against Rbpms and Gap43. *β*-actin served as loading control. Quantification by densitometry of retinal lysates between groups for (**b**) Rbpms, *n*=5–8, (**c**) Gap43, *n*=3, and of ON lysates for (**d**) Rbpms, *n*=3, and (**e**) Gap43, *n*=5–6, normalized to *β*-actin. Data are presented as mean±S.E.M. Statistical significance between treatment conditions determined by unpaired Student's *t*-test, *P< 0.05, **P<0.01, ***P<0.001

To further confirm the beneficial effects of hNRN1 after ONC, retinas and ONs were examined 28 dpc for Rbpms and Gap43 expression by western immunoblotting. Retinal protein lysates revealed increased expression of Rbpms and Gap43 in retinas after AAV2–hNRN1 injection compared with the ONC control AAV2–GFP retinas (Figure 7a). Quantification of Western blots revealed a significant 73% increase (*P*<0.001) in Rbpms expression in AAV2–hNRN1-treated retinas (0.26±0.01, *n*=8) compared with the AAV2–GFP controls (0.15±0.02, *n*=5) (Figures 7a and b). The regenerative marker Gap43 also showed a significant 36% increase (*P*<0.01) in the experimental AAV2–hNRN1 group (2.15±0.09, *n*=3) compared with the AAV2–GFP group (1.57±0.08, *n*=3) (Figures 7a and c).

Rbpms is predominantly expressed in the RGC soma (retinal lysates) but not in RGC axons (ON lysates) (Figures 7a and d). ON Gap43 expression significantly increased 100% (*P*<0.05) in the AAV2–hNRN1 group (0.32±0.04, *n*=6) *versus* the control AAV2–GFP group (0.16±0.02, *n*=5) (Figures 7a and e). The increase demonstrated the promotion of regeneration within the axons of the RGCs.

### AAV2–hNRN1 sustains RGC function after axonal trauma

**Figure 8:**
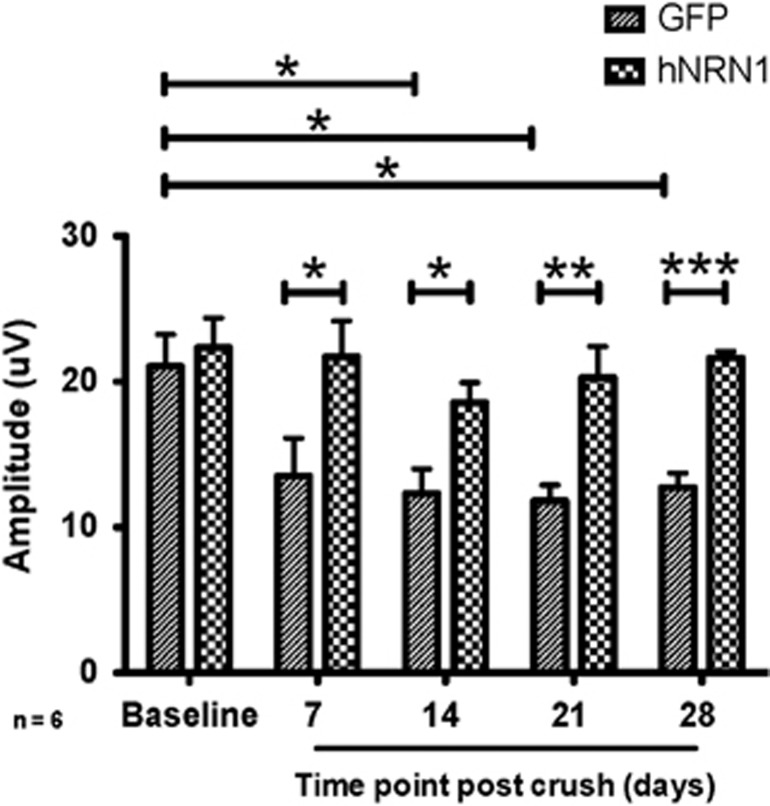
AAV2-CAG–hNRN1 induced sustained RGC function after crush. Animals were intravitreally injected with either AAV2–GFP or AAV2–hNRN1. Two weeks later, animals were subjected to ONC and fERG analyzed at baseline (before crush) and at 7, 14, 21 and 28 days post crush. AAV2–hNRN1 sustains RGC function after ONC. Data are presented as mean pSTR amplitudes±S.E.M. Statistical significance between treatment conditions determined by unpaired Student's *t*-test and across time points determined by one-way ANOVA-Tukey, *post-hoc* test, *P<0.05, **P <0.01, ***P<0.001, *n*=6

The baseline positive scotopic threshold response (pSTR) amplitudes were recorded 10 days after IVT. The baseline pSTR values were similar in the AAV2–GFP- (21.05±2.20 *μ*V, *n*=6) and AAV2–hNRN1- (22.33±2.05 *μ*V, *n*=6) injected mice prior to ONC. Compared with the control GFP group, all the hNRN1-injected mice maintained pSTR amplitudes similar to the initial baseline throughout the 28-day time course after ONC (Figure 8). At 7 dpc, a significant decrease (61%, *P*<0.05) in amplitude was observed in the control group (13.51±2.62 *μ*V, *n*=6) compared with the hNRN1-treated group (21.71±2.46 *μ*V, *n*=6). At 14 dpc, the average amplitude in the hNRN1 group (18.56±1.37 *μ*V, *n*=6) was still significantly greater (51%, *P*<0.05) than the control group (12.31±1.71 *μ*V, *n*=6). At 21 dpc, pSTR amplitudes were significantly decreased (72%, *P*<0.01) in the control group (11.80±1.09 *μ*V, *n*=6) *versus* the hNRN1-treated group (20.26±2.14 *μ*V, *n*=6) group. This difference was still maintained at 28 dpc where a highly significant decrease (70%, *P*<0.001) was observed in the control group (12.73±0.99 *μ*V, *n*=6) in contrast to the hNRN1-treated group (21.63±0.45 *μ*V, *n*=6) (Figure 8). Representative amplitudes and graphs of each time point are presented in Supplementary Figure S4. The protection of RGCs with NRN1 treatment sustained RGC function as assessed by flash electroretinography (fERG). This suggests that the surviving RGCs elicit an effective functional response that maintains amplitudes similar to baseline values.

## Discussion

During early developmental embryonic stages, *Nrn1* promotes survival of neural progenitors and differentiated neurons,^47^ while in later stages it promotes axonal and dendritic growth and stabilization, promoting maturation and formation of synapses.^43, 46, 48^ We demonstrated that recombinant hNRN1 stimulated the survival of axotomized RGCs and increased neurite outgrowth *in vitro*. Further, overexpression of hNRN1 *in vivo* promoted survival of the RGCs and revived the regenerative ability of the injured axons. Finally, the preserved neurons maintained functional light response after ON injury as indicated by fERG.

### Recombinant hNRN1 increases neuroprotection and neurite outgrowth in axotomized RGCs

Functionally, Nrn1 acts as a ligand to the insulin receptor,^60^ and cleavage of the GPI anchor by phospholipase C allows the soluble form to be secreted and activate downstream pathways.^43^ The NGF can induce the transcription and translation of *Nrn1*, which increases neurite outgrowth in cultured rat embryonic hippocampal and cortical neurons,^44^ motor neurons of *Xenopus*^48^ and PC 12 cells.^61^ This increased neurite outgrowth occurs via mitogen-activated protein kinase or phosphatidylinositol-3 kinase activation.^62^

To analyze both the neuroprotective and regenerative effects of NRN1 *in vitro*, we treated RGCs cultured without growth factors with recombinant hNRN1. We investigated RGC survival using Rbpms as a RGC marker. Brn3a is expressed in a smaller subpopulation of RGCs,^63^ while Rbpms is expressed in almost all RGCs.^64^ Our data showed increased RGC survival after NRN1 treatment. In addition, we observed 141% increase in RGC neuritogenesis after application of hNRN1 for 10 days, indicating that prolonged exposure of NRN1 promotes RGC regeneration.

### Neuroprotection and axonal regeneration mediated by AAV2–hNRN1 after axonal trauma

Previously, *in situ* hybridization has shown predominant *Nrn1* expression in the GCL,^45^ and immunoreactivity with NRN1 has also been specifically used to identify RGCs.^65^ In addition, the regenerative role of NRN1 has been reported in previous studies, where it is a downstream effector of neurite outgrowth.^43, 46, 49^ NRN1 enhances the development of motor neuron axon arbors by promoting neuromuscular synaptogenesis and new axon branches.^48^ *Nrn1* mRNA expression shifts predominantly from cell body to axon after nerve crush injury, suggesting the encoding of a growth-associated protein.^66^ Nrn1 is also upregulated after spinal cord injury^24^ and stimulates regeneration of the peripheral neurons.^67^ Silencing of *Nrn1* using siRNA abolished NGF-mediated neurite outgrowth in an experimental diabetic neuropathy model, demonstrating the crucial effect of *Nrn1* in regeneration.^62^ Furthermore, conditional knockout of the *Nrn1* gene in mice delays development, maturation of axons and dendritic arbors, synaptic maturation and effective learning.^68^

Prior studies performed by our group showed downregulation of *Nrn1* within the retina and ON after ONC injury.^51^ In addition, we showed that *Nrn1* exhibits a biphasic pattern of expression after axonal insult. Axotomized RGCs initially increase *Nrn1* expression in an attempt to induce axonal regeneration and overcome obstructed transport mechanisms. These regenerative supportive mechanisms are lost 14 dpc because by then most of the RGCs have been damaged, and the survival of these neurons has progressively decreased.^51^

To test the neuroprotective and regenerative effects of hNRN1, we used an AAV2 delivery vector because of its transduction efficiency and tropism for RGCs.^35, 69^ We found that hNRN1 promotes RGC survival by fivefold at 28 dpc compared with the control GFP vector after axonal trauma. Even though, there is partial protection of RGCs due to hNRN1 overexpression compared with the control GFP group, a prolonged time course would be a more accurate determinant. In addition, designing future experiments using a multifactorial approach would further promote the survival of the RGCs.

NRN1 is a soluble secreted protein, which has been proposed to bind to the insulin receptor. The receptor is present on various cell types within the retina,^70^ and the secreted protein could potentially be beneficial in affecting RGCs in an autocrine manner as well as by other cells in a paracrine manner by having extrinsic effects on the RGCs.

In addition, Gap43 is normally expressed by RGCs during development, and expression is increased transiently after axotomy in mature RGCs.^71^ The expression is further elevated when injured RGCs are stimulated experimentally to regrow their axons.^55^ In our study, there was increased Gap43 expression in RGC axons at 28 dpc. In addition, previously it has been shown that the GPI membrane bound anchor of NRN1 allows it to confer spatial specificity and direct growth promotion.^43^ These data indicate that *Nrn1* may play important roles in neuronal differentiation and survival as well as in neurite outgrowth and axonal regeneration.

### RGC functional activity sustained by AAV2–hNRN1 after axonal trauma

The functional ability of Nrn1 to promote axonal arborization and synaptic plasticity significantly stimulates the rescue of RGCs from apoptosis, prevents regenerative failure and the detrimental loss in RGC function due to ONC. Previous studies have shown that axonal injury following ONC triggers a cascade of intrinsic and extrinsic events resulting in damage to neurons and subsequent regenerative failure^1, 2, 3, 4, 5^ causing RGC apoptosis^72^ and functional defects.^73^ To address the functional deficit of RGCs after crush, we analyzed the effects of hNRN1 on preserving RGC function following ON injury. There was significant increase of 70% in the pSTR amplitude of the NRN1 group compared with the control GFP group at 28 dpc. The maintenance of RGC function by NRN1 treatment was comparable to the pre-ONC baseline further emphasizing the beneficial effects of NRN1 to protect RGCs, enhance regeneration and maintain RGC function. Application of neurotrophic factors such as BDNF has shown enhanced RGC survival and function in other axonal injury models.^74^ In addition, other studies have addressed RGC survivability and axonal regeneration with CNTF and other growth factors,^35, 36^ but most trophic factors affect neuronal survival and regeneration differentially. Nrn1 exhibits recovery from trauma significantly both *in vitro* and *in vivo* which includes RGC survival, axonal regeneration and sustained RGC function without co-deletion of crucial genes such as phosphatase and tensin homolog (*Pten*) and *Socs3* ^38^ or deletion of *Klf4*.^29^

After the initial insult of axotomy, degenerative pathways ensue and during this phase of degeneration, it is crucial for cells to overcome cell death mechanisms and potentiate neurite extensions for survivability and accurate neuronal targeting. *Nrn1* gene therapy was aimed to increase survival and regeneration of axotomized RGCs both *in vitro* and *in vivo.*

Even though oncomodulin and other factors produced by macrophages have been shown to be essential mediators for significantly promoting axonal growth and survival of axotomized RGCs *in vitro* and *in vivo*, oncomodulin requires additional agents, which include mannose to elevate cAMP for stimulating axon outgrowth from RGCs.^75^ Further, intraocular inflammation, cAMP elevation and PTEN deletion exert combinatorial effects leading to regeneration of 1% of the axons past the optic chiasm into the thalamus after ON injury.^76^ Long-distance targeting was achieved to the visual centers of the brain and partial restoration was observed for optomotor response, depth perception and circadian photoentrainment. However, an inflammatory response in the eye could damage other tissues like the lens and retina, and long-term deletion of *pten*, a tumor-suppressor gene, could be detrimental clinically.^77^ In addition, previously, CNTF has been shown to induce regeneration of severed optic axons up to and beyond the optic chiasm, but this caused aberrant growth and misguidance of the axons.^39^

Although our experiments were crucial in identifying *NRN1* as a gene target that could address the different facets of neurodegeneration and regenerative failure, similar experiments need to be performed to test correct innervation of traumatized axons to target neurons over an extended time course. In addition, these studies have been only performed in the ONC model and could be further extrapolated to other CNS trauma models as well as neurodegenerative models.

The ONC model is an acute trauma model and the insult is quite extensive with significant death of RGCs observed as early as 14 dpc.^51^ To ensure that the beneficial effects of NRN1 would induce RGC survival and prevent apoptosis, the RGCs were transduced with AAV2–hNRN1 2 weeks before crush. This experimental design enabled effective transduction of RGCs and sufficient expression of this transgene for promoting survival. To evaluate the true potential of clinical application, the effects of NRN1 need to be investigated after injury. Local application of exogenous NRN1 has been shown to promote axonal regeneration and recovery in locomotor function in rats after acute spinal cord injury.^67^ Similarly, in future experiments recombinant NRN1 could be directly applied to the site of CNS injury. Encapsulated human cells genetically modified to secrete NRN1 could be implanted in regions of CNS trauma to inhibit neurodegeneration. The outer membrane of the semipermeable encapsulated cell implant would permit NRN1 to reach the target area and enable application of the treatment after CNS trauma, allowing the exploration of NRN1 overexpression on survival, regeneration and RGC function after injury.

In conclusion, our study discovered a distinct neuroprotective and regenerative strategy to prevent RGC degeneration. Overexpression of hNRN1 delayed RGC apoptosis, regenerated injured axons and maintained RGC function in an ON injury model.

## Materials and Methods

### Animals

BALB/cJ mice aged 2–4 months were utilized for all the experiments and were obtained from the Jackson Laboratories (Bar Harbor, ME, USA). The mice were housed and maintained in a 12-h light/dark cycle and fed *ad libitum*. All procedures were performed in accordance with the Association for Research in Vision and Ophthalmology Statement on the Use of Animals in Ophthalmic and Vision Research and the University of North Texas Health Science Center (UNTHSC) Institutional Animal Care and Use Committee regulations.

### Retinal cell culture

Isolation and culture of retinal cells was modified from a previously reported protocol.^78^ Briefly, retinas from postnatal day p5–p7 BALB/cJ mice were dissociated with papain solution (2 mg papain per ml (Sigma, St. Louis, MO, USA), 0.4 mg/ml DL-cysteine (Sigma), and 0.4 mg/ml bovine serum albumin (Sigma) in neurobasal medium (Gibco/Invitrogen, Carlsbad, CA, USA)), for 5 min at 37 °C. Dissociated retinas were further dispersed using a 1000 *μ*l pipettor and RGC culture medium^78^ (Neurobasal/B27 medium with 100 U/ml penicillin (Sigma), 100 *μ*g/ml streptomycin (Sigma), 1 mM pyruvate (Gibco/Invitrogen), 2 mM glutamine (Gibco/Invitrogen), 5 *μ*g/ml insulin (Sigma), 100 *μ*g/ml transferrin (Sigma), 100 *μ*g/ml bovine serum albumin (Sigma), 60 ng/ml progesterone (Sigma), 16 *μ*g/ml putrescine (Sigma), 40 ng/ml sodium selenite (Sigma), 40 ng/ml thyroxine (Sigma), 40 ng/ml tri-iodothyronine (Sigma), 50 ng/ml BDNF (Biosource, Camarillo, CA, USA), 10 ng/ml CNTF (Biosource), 10 ng/ml basic fibroblast growth factor (bFGF; Biosource), 5 *μ*M forskolin (Sigma) and 1% fetal calf serum (Atlas Biologicals, Fort Collins, CO, USA)) was added to neutralize the papain. The supernatant was discarded after centrifugation (3000 r.p.m.) to remove any extra papain. RGC medium was added to the pellet and cells were dispersed into a single cell suspension by pipetting. Cells were cultured on poly-D-lysine- and laminin-coated eight-well chambered culture slides (BD Biosciences, San Jose, CA, USA) and incubated in 5% CO_2_/95% air at 37 °C in a humidified incubator. Cells were maintained in two different mediums: medium+GFs (RGC medium with BDNF, bFGF, CNTF and forskolin) or medium+NoGFs (RGC medium with no growth factors) for various treatments.

### Characterization of retinal cell cultures

Dissociated retinal cells were cultured on the chamber slides for 6 days in medium+GFs and fixed in 4% paraformaldehyde (PFA) for 30 min at room temperature. After fixation, the cells were blocked with SuperBlock Blocking Buffer (Fisher Scientific, Chicago, IL, USA) at room temperature for 1 h. Primary antibodies (Supplementary Table S1) were diluted in SuperBlock and incubated overnight at 4 °C. Wells were washed and incubated with AlexaFluor/TRITC secondary antibody (Supplementary Table S1) for 1 h at room temperature. Slides were then rinsed again and mounted with ProLong Gold anti-fade reagent with DAPI (4′,6-diamidino-2-phenylindole, dihydrochloride; Molecular Probes, Grand Island, NY, USA). Images were captured at × 400 magnification using a Nikon Eclipse Ti-U Microscope (Nikon, Melville, NY, USA) with the Nuance Multispectral imaging system (PerknElmer, Inc. Hopkinton, MA, USA) and analyzed using Adobe Photoshop CS5 (Adobe Systems Incorporated, San Jose, CA, USA) software. Images were acquired from random fields of view for every well, and the procedure was repeated in independent culture experiments (eight images/well, *n*=8). Mean number of Brn3a- and Rbpms-positive RGCs were counted and quantified over total number of DAPI cells per image and data are presented as mean±S.E.M.

### Recombinant *Nrn*1 treatment—*in vitro* RGC survival and neurite outgrowth

Dissociated retinal cells were cultured on the chamber slides for 6 days in culture medium (medium+GFs). They were then switched to medium+NoGFs with or without hNRN1 (200 ng/ml). Medium was replaced every 48 h for the next 10 days. After 10 days, the cells were fixed with 4% PFA for 30 min. IHC staining was performed for Rbpms (for RGC survival, *n*=6–7) or Rbpms and Nefl (for neurite outgrowth, *n*=15) and slides were mounted with anti-fade reagent containing DAPI. The RGC survival and neurite outgrowth experiments were performed independently.

For each experiment (survival and neurite outgrowth), images were captured and analyzed at × 400 magnification by fluorescent microscopy for control and treatment conditions (five images/well for survival and four images/well for neurite outgrowth). Each experiment was independently repeated at least three times. Masked image capture and analysis were performed to remove any bias as to the treatment condition. Cell counts were analyzed using Adobe Photoshop software. Data are presented as mean±S.E.M. of replicate wells. RGC survival was evaluated by counting Rbpms-positive cells over total number of DAPI-positive cells. For neurite outgrowth studies, RGCs were photographed and neurite length was determined using ImageJ software (Research Services Branch, Bethesda, MD, USA) with the NeuriteTracer plugin (Fournier Lab, Montreal, QC, Canada). Processing of nuclei and neuron images was compiled using the plugin with normalization and standardization procedures in place. Total neurite length per 39 100 *μ*m^2^ area was calculated for the control and NRN1-treated cells.

### AAV2 production and IVT administration

For AAV2 production, we used a pCMV6-XL5 plasmid (Origene, Rockville, MD, USA) carrying the hNRN1 (NM_016588) cDNA clone. Vector Biolabs (Philadelphia, PA, USA) performed the AAV cis cloning, cis-plasmid preparation, viral packaging, viral purification and GC titration of AAV2-CAG–hNRN1-WPRE. AAV2–GFP with the CAG/CBA promoter was also ordered from Vector Biolabs.

For AAV2 IVT injections, mice were anesthetized by intraperitoneal (i.p.) injection of ketamine (100 mg/kg) and xylazine (10 mg/kg) and 2 *μ*l of AAV2-CAG–GFP or AAV2-CAG–hNRN1 (10^10^ GC) was injected into the left eye using a Hamilton syringe (Sigma-Aldrich, St. Louis, MO, USA). The contralateral eye was left untreated. To study transduction efficiency, single IVT AAV2 injections were administrated and mice harvested at 2, 3, 4 and 6 weeks post injection (*n*=4). For studying RGC survival (*n*=3–7), axonal regeneration (*n*=4–8) and RGC function (*n*=6), single AAV2 injections were administered 2 weeks prior to ONC surgery and mice were harvested or tested at 7, 14, 21 and 28 dpc for RGC survival, RGC function and at 28 dpc for axonal regeneration.

### ONC model

The ON of the left eye was crushed 0.5 mm posterior from the globe for 4 s using the Nickell's technique.^79^ Briefly, mice were anesthetized by i.p. injection of ketamine (100 mg/kg) and xylazine (10 mg/kg). The ON (left) was exposed and crushed intraorbitally using self-closing forceps to ensure reproducibility and constant force. Extreme care was taken not to damage the ocular blood vessels. Indirect ophthalmoscopy was performed to ensure retinal circulation was not compromised.

### Immunohistochemistry

IHC was performed on retina and ON cryo-sections and SC paraffin sections to validate protein expression of Rbpms (RGC survival, *n*=3–7) and NRN1 and GFP (AAV2-induced overexpression, *n*=4). Whole eyes with ONs were harvested and fixed in 4% PFA for 2 h at room temperature. After fixation, the tissue was placed in 20% sucrose overnight at 4 °C and embedded in optimum cutting temperature the next day. Sections (10 *μ*m) were cut using a cryostat (Leica Biosystems, Richmond, IL, USA). Cross-sections of retina were transferred to Superfrost glass slides (Fisher Scientific). Slides were incubated in PBS for 10 min and blocked with SuperBlock Blocking Buffer at room temperature for 1 h. Primary antibodies (Supplementary Table S1) were diluted in SuperBlock. Each slide was incubated with the respective primary antibody and incubated overnight at 4 °C. Sections were then washed and incubated with AlexaFluor secondary antibody (Supplementary Table S1) for 1 h at room temperature. After rinsing, slides were mounted with ProLong Gold anti-fade reagent with DAPI. Sections were observed and captured using a Nikon Eclipse Ti-U Microscope containing the Nuance Multispectral imaging system and analyzed using Adobe Photoshop CS5 software.

### Survival counts of the RGCs *in vivo*

RGC survival counts were determined by counting Rbpms-positive cells in a masked manner from six retinal sections from ora serrata to ora serrata through the ON head. To perform survival counts, each Rbpms-positive cell was merged with DAPI and counted individually to represent number of RGCs. The level of fluorescence intensity per cell was not utilized as a marker of expression. Cell counts from all six sections were averaged per retina and the mean of all retina counts determined for both experimental conditions: control (AAV2–GFP) and treatment (AAV2–hNRN1) for each time point post crush (7,14, 21 and 28 dpc, *n*=3–7). The data (mean±S.E.M.) were analyzed by comparing both the groups: treatment (AAV2–hNRN1) *versus* control (AAV2–GFP).

### RNA *in situ* hybridization

RNA *in situ* hybridization was performed to verify effective transduction of AAV2–GFP and AAV2–hNRN1 (*n*=4). Cryo-fixed retina cross-sections were subjected to protein digestion using proteinase K stock (Panomics, Santa Clara, CA, USA) diluted 1 : 100 for 20 min at room temperature. *In situ* hybridization was performed using type 1 probes for hNRN1 (VAI-15422) designed by Panomics following manufacturer's protocols. Briefly, probes were diluted in 1 : 50 in hybridization buffer and sections incubated at 40 °C for 3 h and then overnight at room temperature. The sections were then washed and hybridized in succession after each of the following treatment: PreAmp1 QF (1 : 100) at 40 °C (25 min), Amp1 QF (1 : 100) at 40 °C (15 min), Label Probe AP (1 : 1000) at 40 °C (15 min). Sections were finally incubated with AP Enhancer solution (Affymetrix, Inc., Santa Clara, CA, USA) for 5 min at room temperature and Fast Red substrate (chromogenic dye) at 40 °C (30 min). Slides were washed and mounted with ProLong Gold anti-fade reagent with DAPI. Sections (three sections/mouse, *n*=4) were observed and captured at × 400 magnification using a Nikon Eclipse Ti-U Microscope containing the Nuance Multispectral imaging system and analyzed using Adobe Photoshop CS5 software.

### Retinal flat mounts

Gap43 and Nefm labeling of RGC axons within the GCL was performed with a modified flat-mounting protocol.^73, 80^ Two weeks after IVT injection of either AAV2–GFP or AVV2–hNRN1, ONC was performed. At 28 dpc, mice (*n*=5 per group, AAV2–GFP and AAV2–hNRN1) were killed, eyes enucleated and fixed in 4% PFA (Electron Microscopy Sciences, Hatfield, PA, USA) for 2 h. Post fixation, retinas were carefully dissected and washed several times with PBS. Pre-treatment with 0.3% Triton X-100 was done for an hour at room temperature. Retinas were then soaked in blocking buffer (10% goat serum, 0.3% Triton X-100, 60 min, room temperature) and then incubated with primary antibodies (Supplementary Table S1) diluted in 10% goat serum+0.3% Triton X-100 using a modified protocol with incubation overnight at room temperature. The next day, retinas were left at 50 °C for 4 h and washed multiple times with PBS and incubated with AlexaFluor secondary antibody (Supplementary Table S1) diluted in 0.1% Triton X-100 with gentle agitation overnight at 4 °C. After rinsing, retinas were flat-mounted RGC side up with ProLong Gold anti-fade reagent with DAPI. Images at x400 magnification were observed and captured using a Nikon Eclipse Ti-U Microscope containing the Nuance Multispectral imaging system and analyzed using Adobe Photoshop CS5 software.

### ON 3D rendering

To show CTB-594 transport through the ONs post crush, mice overexpressing either GFP or hNRN1 were injected intravitreally with 2 *μ*l of CTB conjugated with AlexaFluor 594 (1 *μ*g/ul) at 26 dpc (AAV2–GFP, *n*=4, AAV2–hNRN1, *n*=8). The CTB conjugate was taken up by RGCs and anterogradely transported. To visualize CTB-594 labeled axons in whole ONs, mice were then killed 48 h post injection and ONs cleared using a previously published protocol.^81^ Briefly, a 2-h 4% PFA fixation was performed, ONs were rinsed twice in PBS and dehydrated in increasing concentrations of ethanol (50, 80 and 96%) for an hour at room temperature with gentle agitation and left overnight in 100% ethanol. To remove any traces of water, ONs were then placed in 100% hexane for 2 h at room temperature and a clearing solution composed of mixture of benzyl alcohol and benzylbenzoate (1 : 1; Sigma-Aldrich) was rapidly added post hexane treatment. The white ONs turned transparent within 30 s to 1 min. Whole ONs were mounted in clearing medium before imaging. Image stacks were captured using a confocal Zeiss LSM 510 Meta system (Microscope is Axiovert 200 M, Carl Zeiss, Jena, Germany) × 400 objective (NA: 1.2). Axons were scanned using three-dimensional (3D) reconstruction of three consecutive Z-stacks starting from the crush site and images stitched using the Zen (Thornwood, NY, USA) software. The resulting macro-stacks were exported to ImageJ and Zen to create 3D projections. Fluorescence intensity was measured using ImageJ plugin with normalization and standardization procedures in place. The data (mean±S.E.M.) were analyzed by comparing both the groups: treatment (AAV2–hNRN1) *versus* control (AAV2–GFP).

### Western blot assay

For retinal and ON lysate preparation, mice were killed, eyes enucleated and retinas and ON were carefully dissected. Each isolated tissue sample was homogenized in MPER lysis buffer (Fisher Scientific, Pittsburgh, PA, USA) with 1% protease inhibitor cocktail (Pierce Technology, Rockford, IL, USA) and the lysate supernatant collected after centrifugation at 5000 r.p.m. (10 at 4 °C). Protein concentration was determined by the BioRad Dc Protein Assay Kit (Bio-Rad Laboratories, Hercules, CA, USA). Proteins were separated on 15% polyacrylamide gels with 35 *μ*g protein applied per lane, and transferred to nitrocellulose membranes (Amersham, Buckinghamshire, UK). Membranes were blocked (5% milk in TBST buffer) and incubated overnight at 4 °C with primary antibody (Supplementary Table S1). The membranes were washed with TBST and probed with horseradish peroxidase-conjugated secondary antibody (Supplementary Table S1) in 5% non-fat milk in TBST for 1 h at room temperature. Proteins were detected using the ECL reagent SuperSignal West Femto Maximum Sensitivity Substrate (Pierce Biotechnology) and a FluorChem 8900 imager (Alpha Innotech, Santa Clara, CA, USA). Immunoreactivity was quantified using the FluorChem spot densitometry software.

### Full field fERG recording

Mice were dark-adapted overnight prior to ERG recording. The next day all the procedures were performed in a dark room with a dim red safe light as needed. The mice were anesthetized during the recording procedure by Isoflurane (Bulter Schein Animal Health, Dublin, OH, USA) inhalation for precise quantitative comparisons as reported previously.^82^ Their body temperature was maintained at 37 °C by a heating pad and monitored using a rectal probe. A handheld multi-species-electroretinograph (ERG) unit (Ocuscience, Kansas City, MO, USA), with thread electrodes was utilized to perform the full field fERG. Pupils were dilated with 2.5% phenylephrine ophthalmic eye drops. The thread electrodes were carefully placed on top of each cornea. The cornea was moistened with 2.5% hypromellose ophthalmic solution (Arkon, Lake Forest, IL, USA) and covered with a 2.5 mm clear contact lens. The corresponding stainless steel reference electrode was placed under the skin in the area below each eye. Another grounding needle electrode was inserted subcutaneously above the tail. Simultaneous bilateral ERG recordings were performed on the mouse eyes.

The light stimuli impulse was generated in a Ganzfeld dome with light emitting diodes. The scotopic ERG responses were recorded by stimulating the retina with a flash intensity of 3 × 10^−5^cd.s/m^2^. Responses from 30 flashes with 2-s intervals were averaged. The amplitudes of pSTRs were measured from the baseline to the positive peak of each waveform and latency measured by time-to-peak major positive deflection. The data (mean±S.E.M.) were analyzed by comparing both the groups: treatment (AAV2–hNRN1) *versus* control (AAV2–GFP).

### Statistical analysis

One-way ANOVA followed by Tukey *post-hoc* test was utilized to analyze intragroup differences across time points and unpaired Student's *t*-test was used to analyze data between two groups. Data are presented as mean±S.E.M. and a *P*<0.05 was considered statistically significant.

## Abbreviations

AAV: adeno-associated-virus
bFGF: basic fibroblast growth factor
BDNF: brain-derived neurotrophic factor
Brn3a: brain-specific homeobox domain protein/POU domain protein 3A
CNTF: ciliary neurotrophic factor
CNS: central nervous system
CTB: cholera toxin B
dpc: days post crush
fERG: flash electroretinography
GAP43: growth-associated protein 43
GCL: ganglion cell layer
GFP: green fluorescent protein
GPI: glycophosphatidylinositol
IHC: immunohistochemistry
IVT: intravitreal injection
NGF: nerve growth factor
NRN1: Neuritin 1
NT-4: neurotrophin-4
ON: optic nerve
ONC: optic nerve crush
PFA: paraformaldehyde
PN: peripheral nerve
pSTR: positive scotopic threshold response
Rbpms: RNA binding protein with multiple splicing
RGC: retinal ganglion cell
SC: superior colliculus
