# Supplementary figures and images for: Neuritin 1 promotes retinal ganglion cell survival and axonal regeneration following optic nerve crush
Source: Cell Death Dis. 2015 Feb 26;6(2):e1661–. doi: 10.1038/cddis.2015.22 (PMC4669798; doi:10.1038/cddis.2015.22)

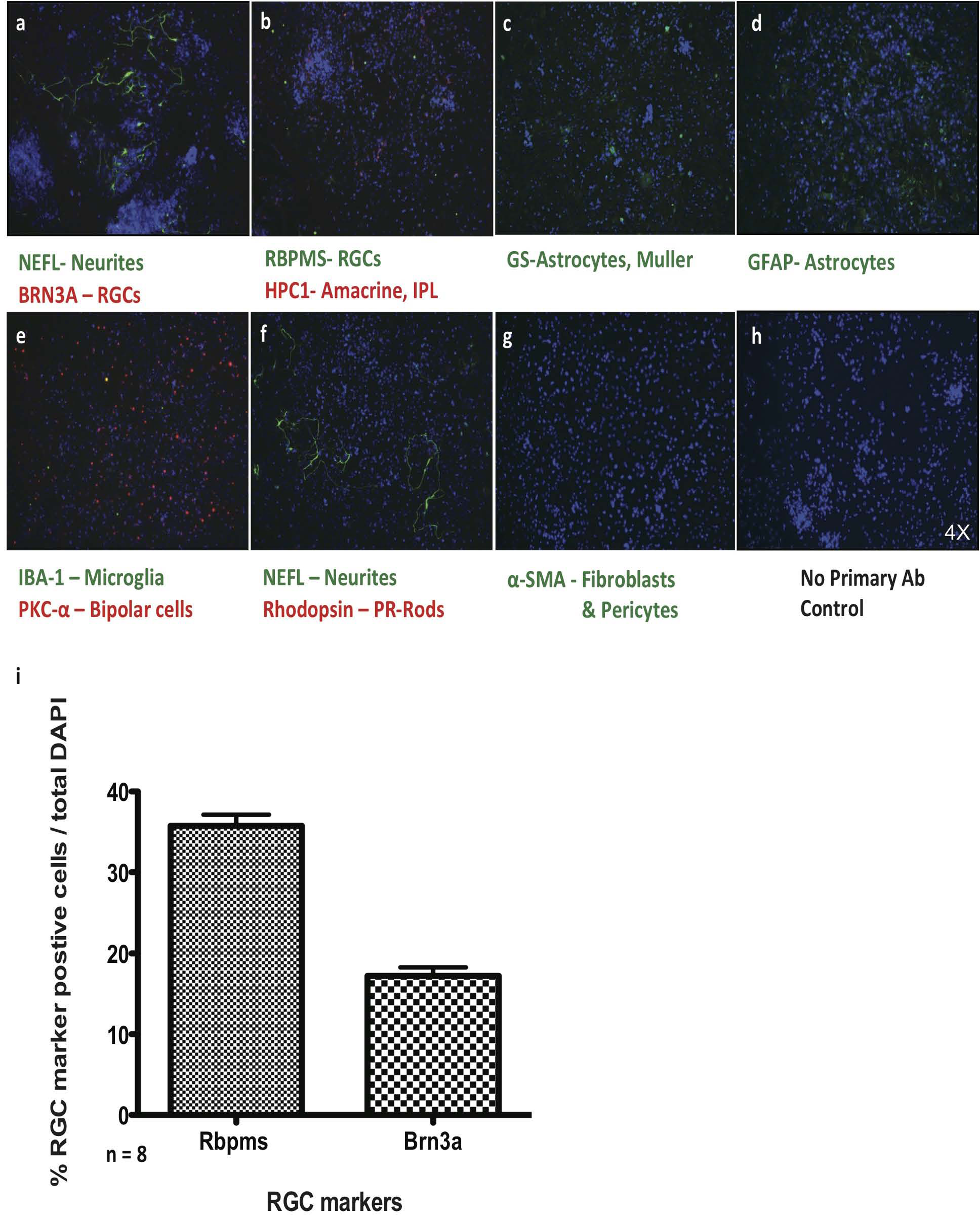

Supplement: Supplementary Figure S1 [file cddis201522x1.tif]

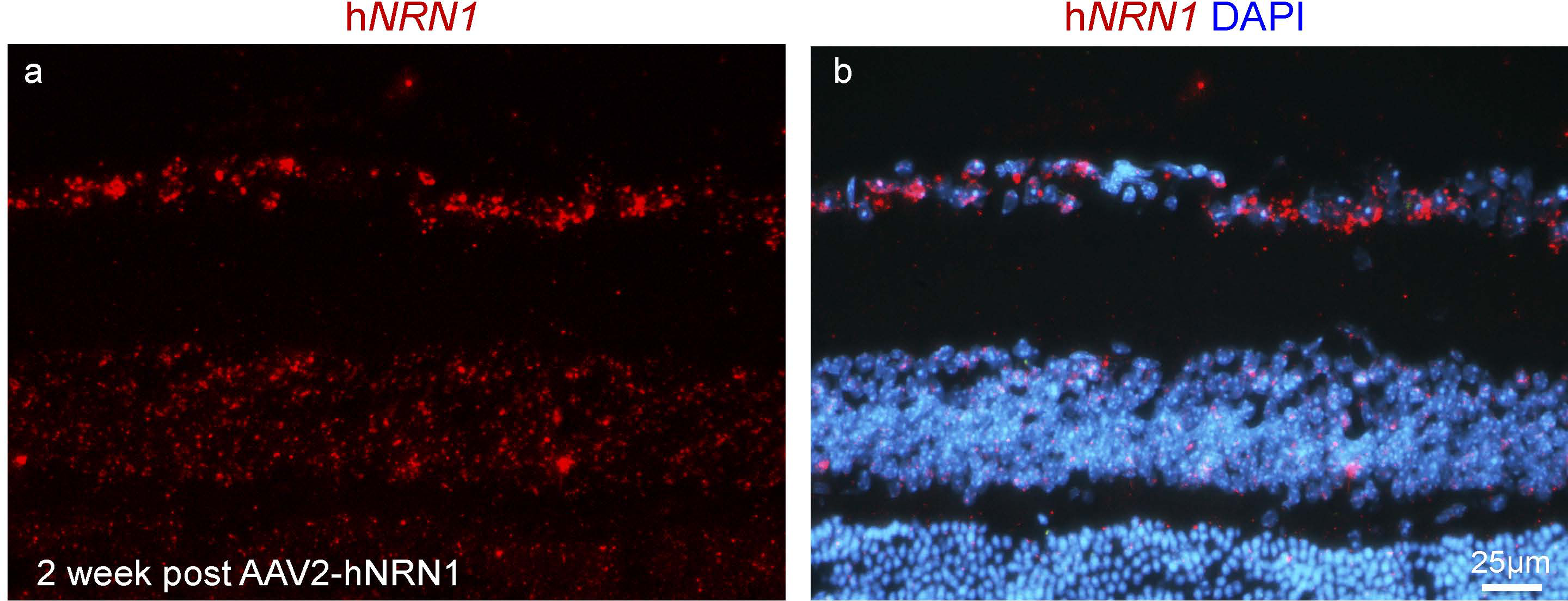

Supplement: Supplementary Figure S2 [file cddis201522x2.tif]

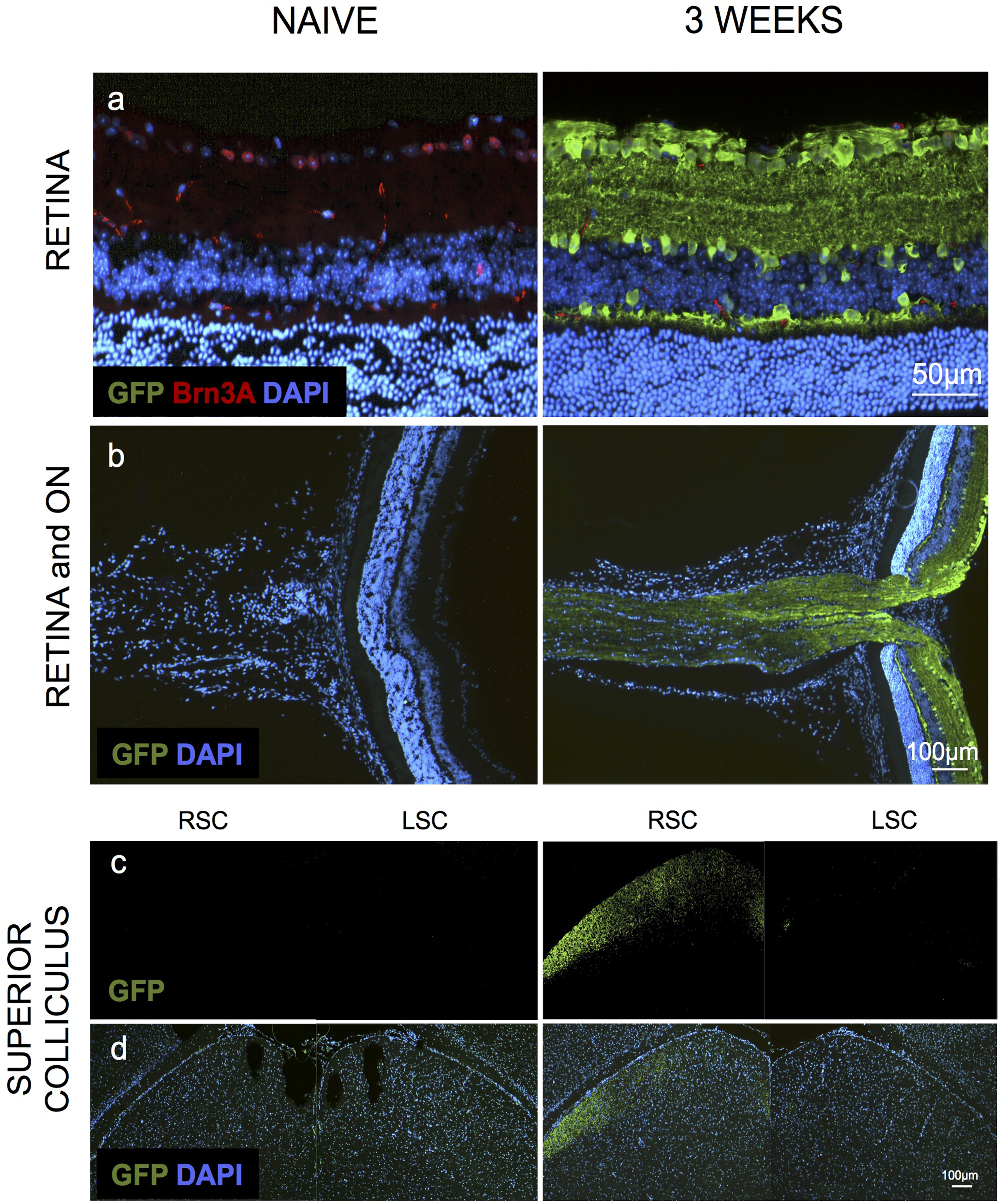

Supplement: Supplementary Figure S3 [file cddis201522x3.tif]

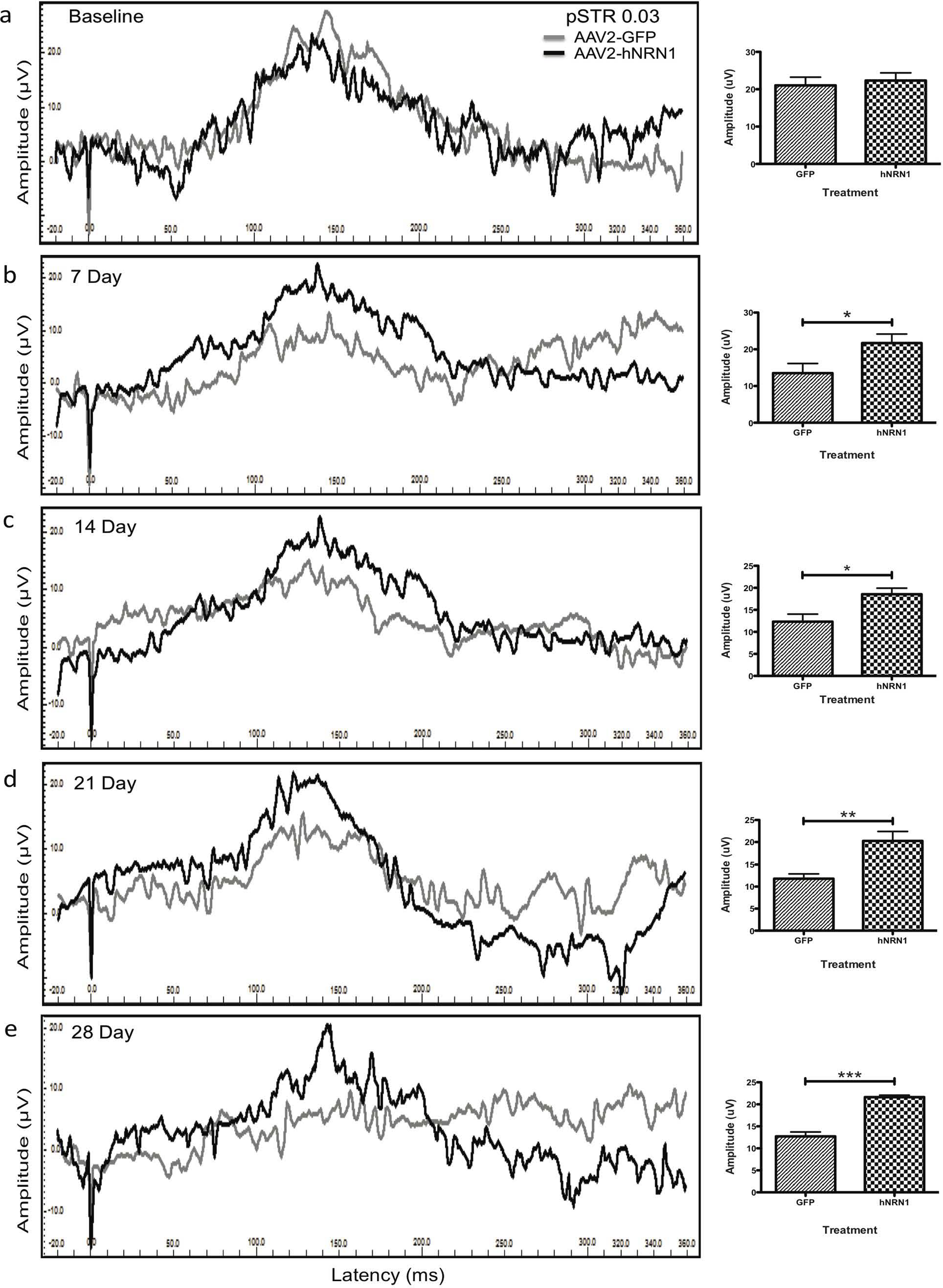

Supplement: Supplementary Figure S4 [file cddis201522x4.tif]

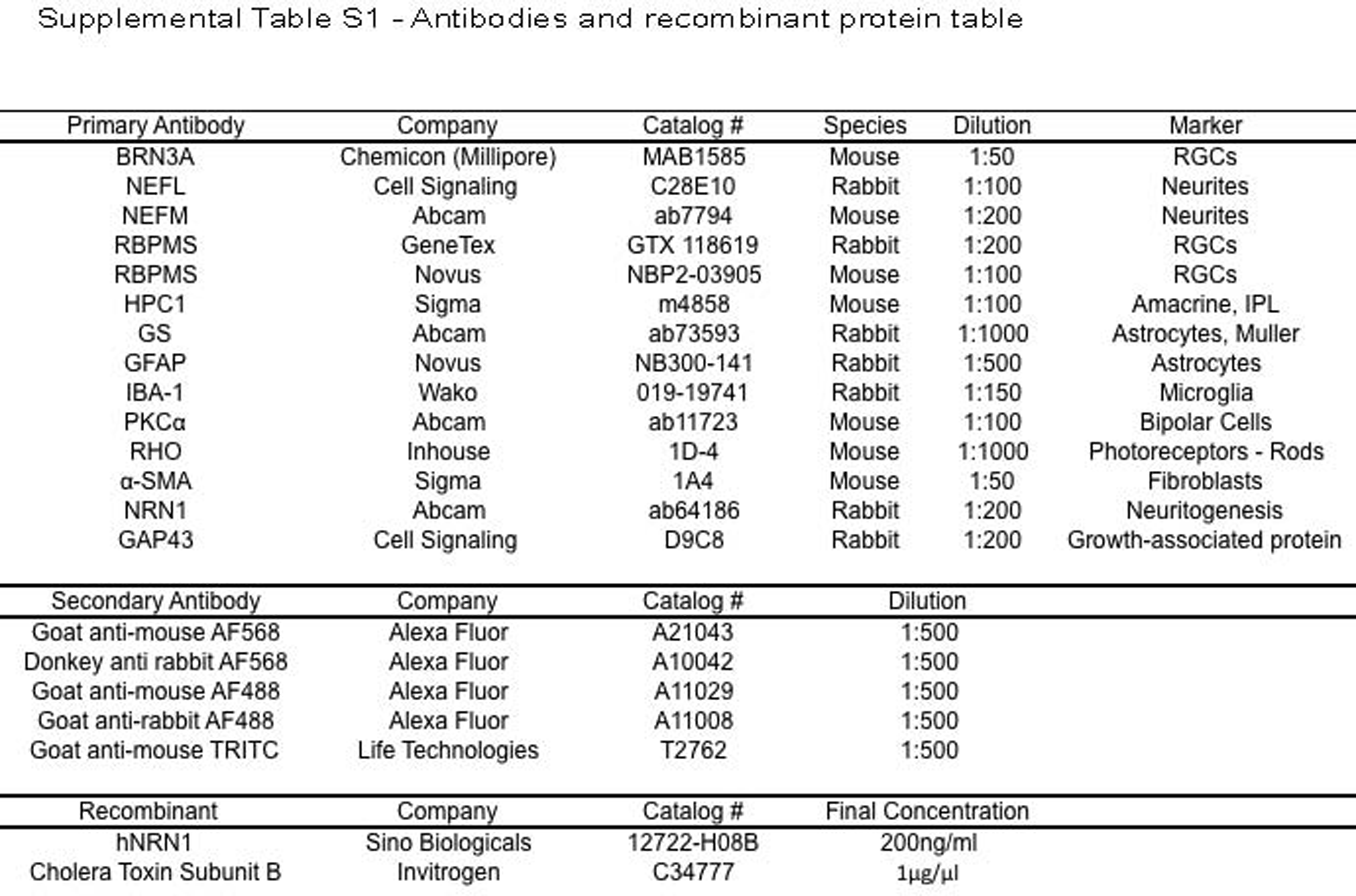

Supplement: Supplementary Table S1 [file cddis201522x5.tif]
