# Supplementary material for: Neuritin 1 promotes retinal ganglion cell survival and axonal regeneration following optic nerve crush
Source: Cell Death Dis. 2015 Feb 26;6(2):e1661–. doi: 10.1038/cddis.2015.22 (PMC4669798; doi:10.1038/cddis.2015.22)
Supplement: Supplementary Information [file cddis201522x6.doc]

**Supplemental Figure S1.jpg**

**Characterization of cells and quantification of RGCs within the *in vitro* mixed retinal cell culture**. Photomicrographs were captured at 400X original magnification. Values represent the mean of 8 wells from three independent experiments per group. Data presented as mean ± SEM, n=8.

**Supplemental Figure S2.jpg**

**Increased retinal neuritin mRNA expression after AAV2 mediated *NRN1* overexpression.** BALB/cJ mice received intravitreal injections of AAV2-hNRN1. *In situ* hybridization performed on retinal sections probed for *NRN1.* Retinal sections from 2 weeks (a) *NRN1*, (b) *NRN1* and DAPI. Red= *NRN1*, blue = DAPI. Scale bar = 25μm, n=4.

**Supplemental Figure S3.jpg**

***In vivo* expression of GFP using AAV2 viral vectors.** BALB/cJ mice received intravitreal injections of AAV2-GFP. Retinas, ONs and SC harvested at naïve and 3 weeks after injection. Fluorescence micrographs showed over-expression of GFP (3 tissues). Red= Brn3a, blue = DAPI. Scale bar = 50μm, 100μm, n=4.

**Supplemental Figure S4.jpg**

**Amplitudes and graphs of pSTRs after AAV2 mediated GFP or hNRN1 injections *in vivo*.** Animals were intravitreally injected with either AAV2-GFP or AAV2-hNRN1. Two weeks later, ONC performed and pSTRs analyzed at baseline, 7, 14, 21 and 28 dpc. Mean pSTR amplitude ± SEM graphically presented, * p value<0.05, n=6.

**Supplemental Table S1.jpg**

**Antibodies and recombinant protein table.** Information and dilutions of primary antibodies, secondary antibodies and recombinant protein presented in tabular format.
